# Food Exchange List and Dietary Management of Non-Communicable Diseases in Cultural Perspective

**DOI:** 10.12669/pjms.335.13330

**Published:** 2017

**Authors:** Mahnaz Nasir Khan, Samia Kalsoom, Ayyaz Ali Khan

**Affiliations:** 1 Mahnaz Nasir Khan, Assistant Professor, Kinnaird College for Women, Lahore, Pakistan; 2 Prof. Dr. Samia Kalsoom, Government College of Home Economics, Lahore, Pakistan; 3 Prof. Dr. Ayyaz Ali Khan, Shaikh Zayed Post Graduate Medical Institute, Lahore, Pakistan

**Keywords:** Diet, Exchange List, Non-Communicable Diseases

## Abstract

This review focuses at highlighting the importance of Food Exchange List in cultural perspective, as an effective dietary tool to help individuals’ manage their dietary modifications in relation to non communicable diseases whilst specifying measures that can help improve the quality of Food Exchange Lists for combating various non communicable diseases and addressing adherence related issues to specialized diets. A search was done using PubMed & Google Scholar till June 2016. Search terms used were food exchange list AND disease, diet AND non-communicable diseases. We included only studies that discussed Food Exchange List (FEL) in relation to non-communicable diseases; in addition to factors like cultural relevance and adherence.

Out of the 837 papers accessed 57 were identified as relevant to the Food Exchange List, out of which 39 papers were focused to the concept and development of the Food Exchange List. Only 18 discussed FEL in relation to non communicable diseases and were thus included in the review.

Food exchange list is a user friendly tool for dietary modification due to disease. This tool may help to customize meals for people as it provides information regarding various food items in different groups. This tool is helpful in reducing blood & plasma glucose levels, maintaining lipid profile & effectively combating other diet related diseases & those ailments in which diet plays a significant role in maintenance & prevention from reoccurrences. However, better management and adherence to modified diets for non communicable diseases can be ensured by keeping cultural relevance under consideration before using Food Exchange Lists for such diseases.

## INTRODUCTION

Diet and nutrition are important factors in the promotion and maintenance of good health throughout the entire life; their role as determinants of chronic non communicable diseases is well established and they therefore occupy a prominent position in preventive medicine.^1^ According to World Health Organization (WHO, 2015)² 38 million people die each year from non-communicable diseases (NCDs). The percentage of NCDs is projected to double by 2020, accounting for 75% of all deaths worldwide, out of which 71% of deaths will be due to ischemic heart disease (IHD), while stroke will be responsible for 75% and 70% of the deaths will result from diabetes in developing countries.^3^ Food and Agriculture Organization (FAO) and World Health Organization (WHO) has thus supported the development of guidelines formulated on scientific basis to fight against diet-related public health problems.^4^

The treatment and management of these non communicable diseases seems easy and approachable i.e. through dietary modification but literature review on dietary patterns in relation to nutrient adequacy, demographic variables and health outcome^5^ reveal that this task is not as uncomplicated as it seems. Planning a healthful diet is not simple as it not only involves the principles of a healthy diet, but also takes into consideration factors influencing food choices which include personal preferences, habits, ethnic heritage and tradition.^6^ Consideration of these factors while planning a diet may increase the commitment of consumers for whom the diet has been planned. Hence, having a meal planning tool that considers all previously mentioned factors is a must in promoting better nutrition both at an individual and community level.^7^

Food Exchange List (FEL) is a user friendly tool which was developed to help individuals to aid healthy eating habits and follow a specific diet plan. This may be a helpful supplementary strategy when helping patients prevent or manage non-communicable diseases that are affected by diet especially those with diabetes.

Numerous studies focusing on the cultural relevance of the food exchange lists validate the viewpoint that quality of Food Exchange Lists for better management of various non-communicable diseases can be improved by developing FELs possessing specific cultural relevance.

## METHODS

A search was done using PubMed & Google Scholar till January, 2016. Search terms used were “food exchange list”, “food exchange list and disease” and “diet and non communicable diseases”. The results were downloaded into END Note.

### Inclusion criteria

We included studies only if their center point was FEL and non-communicable diseases.

**Fig.1:**
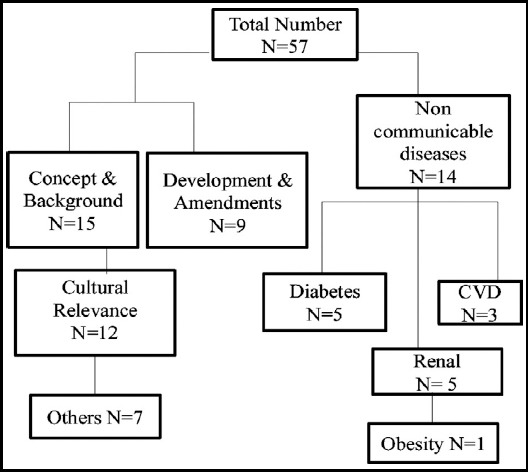
Characteristics of the Included Studies.

We excluded all the studies focusing at dietary intervention and diseases without following any exchange system. Alcohol consumption, life style changes and its relationship with non communicable diseases were also excluded from this review. A schematic representation of the study inclusion process is presented in Fig.1.

## RESULTS

A total of 137 papers were found related to dietary management of non communicable disease; after title and abstract screening 57 papers were related to FEL while only 14 papers fulfilled the inclusion criteria of the study which are presented as Table-I.

**Table-I:** Link between the FEL and Disease.

*Study*	*Type of FEL*	*Disease*	*Study Focus*
A culturally sensitive tool for Mexican people with diabetes: La Manzana de la Salud.^7^	Mexican FEL	Diabetes Mellitus	Emphasized on the importance of cultural sensitivity in FEL and addition of local foods for use in Central America
Differences between analyzed and estimated sodium contents of food composition table or food exchange list.^25^	Korean FEL for hypertensive patients	Hypertension	Focused on the effectiveness of FEL in preparation of Low sodium diets compared to food composition tables
Development of the South African renal exchange lists.^24^	Novel renal exchange list	Chronic Kidney Disease	The novel renal exchange list contained food groups based on protein, potassium and energy content and is currently used for counseling and planning diets for patients with Chronic Kidney
Choose Your Foods: Exchange Lists for Diabetes, 2008: description and guidelines for use.^10^	FEL by American Dietetic Association and American Diabetic Association	Diabetes Mellitus	Emphasized on giving food choices within the same food group in planning a diet for a diabetic distributing carbohydrate intake during the day.
The Fat Portion Exchange List: a tool for teaching and evaluating low-fat diets.^12^	Fat Portion & FEL	obesity, cardio-vascular disease and natural killer (NK) cell activity	Focus was placed on the serving size of fat portion.
Development of exchange lists for Mediterranean and Healthy Eating Diets: implementation in an intervention trial.^17^	Mediterranean FEL	Cardio vascular disease (CVD)	Importance was placed on the diet quality for achieving positive outcomes from dietary intervention
Choose your foods: exchange lists for diabetes: the 2008 revision of exchange lists for meal planning.^11^	FEL by American Dietetic Association and American Diabetic Association	Diabetes Mellitus	Revised and maintained focus on planning diet for diabetic while providing them choices.
Mediterranean alpha linolenic acid rich diet in secondary prevention of coronary artery disease.^14^	Mediterranean FEL	Coronary artery disease (CAD).	Intake of omega 3 fatty acid was highlighted as secondary prevention of CAD.
Traditional foods: a science and society perspective.^15^	FEL	CVD &CAD	Importance placed on intake of monounsaturated fatty acids
Design of a Mediterranean exchange list diet implemented by telephone counseling.^16^	FEL	CVD &CAD	Strategies of implementation were highlighted
Study	Type of FEL	Disease	Study Focus
Development of exchange lists for Mediterranean and Healthy Eating Diets: implementation in an intervention trial.^17^	Mediterranean FEL	Overweight & obesity	Focused on improving diet quality
Medical Nutrition Therapy Evidence-Based Guidelines for Practice: Nutrition Practice Guidelines for Gestational Diabetes Mellitus.^19^	FEL by American Dietetic Association and American Diabetic Association	Gestational Diabetes Mellitus	Achieving appropriate blood glucose levels by distributing carbohydrate into five- seven meals and snacks
A Simple Meal Plan Emphasizing Healthy Food Choices Is as Effective as an Exchange-Based Meal Plan for Urban African Americans With Type 2 Diabetes.^20^	FEL for African Americans	Diabetes mellitus Type 2	Focused on reducing HbA1c levels through diet
Development and validation of an expedited 10 g protein counter (EP-10) for dietary protein intake quantification.^21^	FEL as a protein counter	Renal	Use of FEL as a protein counter for management of renal disease.
A practical approach to the nutritional management of chronic kidney disease patients in Cape Town, South Africa.^22^	South African FEL for chronic kidney disease	Chronic kidney disease	Culturally sensitive FEL for renal patients.
Southeast Asian renal exchange list.^23^	Southeast Asian Renal FEL	Chronic kidney disease	Culturally sensitive FEL for renal patients.

## DISCUSSION

Recent disease and diet related literature has brought to light that while reviewing the relationship between health, disease and overall diet, a single nutrient should not be focused rather food or food groups should be taken into consideration as free-living individuals eat a combination of foods that provide them a mixture of nutrients.^5^ Interventions should therefore be encouraged for healthy food choices including a balance of macro and micro nutrient content.^8^

Meal planning food exchange list formulated in 1950^9^ is such a tool which allows the interchanging of foods within a particular food group so as to provide flexibility to the consumer, ensuring better adherence to the dietary regime in relation to the management of disease. The food exchange list has undergone five revisions to keep at pace with the current developments in food, nutrition and its relationship to health and is still considered the most appropriate tool for management of non communicable disease like diabetes mellitus^10^ and cardio-vascular disease (CVD).^11^

The quality and quantity of fat has always been related to many NCDs like obesity, cardio-vascular disease and natural killer (NK) cell activity are just a few to mention.^13^ Fat Portion exchange system thus developed as a counseling tool to help individuals follow a low fat diet. This system comprises of a broad catalog of food list that is grouped according to its fat type and fat portion. The serving size of one fat portion equals 5 grams of fat and intake of total fat portions for an individual is calculated according to the total daily caloric requirements.^12^

The activity of natural killer (NK) cells in humans is also linked with fat intake and studies supports that reduced or low intake of dietary fat diet is positively correlated with the increased activity of NK cells.^13^ The type of fat and its effect on NK cell activity also indicate an increase in NK activity between 20% to 50% on low fat and menhaden fish oil [containing both EPA (Eicosapentaenoic Acid) and DHA (Docosahexaenoic Acid).^14^

The focus of total dietary fat intake and its type was further enforced when the Lyon Diet Heart Study showed substantial evidence to support the effect of low fat Mediterranean Diet on cardio vascular disease; it conferred its protective effect for up to four years after the first myocardial infarction.^15^ The Mediterranean diet presents a food model that can be used for a healthy dietary pattern with ease as it offers both taste and flavor to a simple cuisine.^16^ This Mediterranean food exchange list is based on two distinct aspects, that is, a high intake of monounsaturated fats about 30% to 40 % of the total energy intake^17^ and vegetables along with a moderate intake of protein. Adherence to Mediterranean diet regime through counseling is achievable as a six month intervention trail showed an increase in the fruit and vegetable intake from 4.0 to 8.6 servings per day and 48% increase in dietary monounsaturated fat.^18^ Thus, it may be said that both a low Fat Portion exchange system of 1989 and a low fat Mediterranean food exchange list (2014) were equally successful and ended up with the consensus that it is a useful dietary tool for improvement of diet quality and could be used for achieving positive outcomes for intervention.^19^

The food exchange list is being used as a means of providing medical nutritional therapy to diabetics^20^ since 1950 and its fifth revision came with a new title of *Choose Your Foods: Exchange List for Diabetes*.^10^ This tool was designed to assist in translating evidence based nutrition recommendations into healthful eating choices^11^ and distributing carbohydrate intake during the day into five to seven small to moderate meals and snacks^21^ reducing HbA1c levels in individuals with type 2 diabetes.^20^

Quantification of protein is also essential in clinical dietetics, especially while dealing with patients with renal impairment, burn, or malnourished patients. Besides the ADA Food Exchange List^10^ a 10 g protein counter (EP-10) was also developed to expedite the estimation of dietary protein for nutritional assessment and recommendation and it was seen that both EP-10 and ADA-7g are valid clinical tools for protein intake^21^ A Food Exchange List was also fashioned for renal patients living South Africa^24^ and for those belonging to Southeast Asian origin. The novel renal exchange list contained food groups based on protein, potassium and energy content and is currently used for counseling and planning diets for patients with Chronic Kidney Disease since it incorporates varied options and choices within the same food group as well as offers ease of description to the patients in terms of portion size.^25^ The protein quantity of the food was the determination factor of the portion sizes.^24^

High intake of sodium as part of dietary pattern has been associated with increased risk of developing hypertension. The effectiveness of low sodium diet was determined using a diet planned through food exchange list for hypertensive patients and it was found that low sodium diet prepared using exchange list was more effective than the one prepared using food composition tables.^25^

Medical Nutrition Therapy has also played a pivotal role for the treatment and management of metabolic disorders like Phenylketonuria (PKU) and maple syrup urine disease (MSUD) where amino acid metabolism is involved and dietary restriction very early in life to avoid neurological delays and defect. Use of the standard Food Exchange list for the treatment and management of diabetes showed limitation as it was cultural insensitivity and modification to the standard food exchange list was done by addition of local foods so that it could be used in Central America.^7^

Food Exchange List is an appropriate tool for effective nutrition education, intended for improving nutrition knowledge, attitudes and dietary behaviors both at an individual and community level.^26^ However, to ensure that this tool is more effective cultural variations will have to be taken into consideration so as to provide the user with food items with which he or she is more familiar as exact menus and food amounts are a prerequisite to ascertain nutrient adequacy of any dietary regime.^27^

### Improved Quality of Food Exchange List and Adherence to Specific Diets

Dietary Tools that are reassessed and modified to cater new demands have proved to be more effective in relevance to NCD management. Korean FEL was updated in 2001 as the prevailing FEL was complicated and not practical to use even by dietitians.^28^ A proper understanding of the FEL by clinical nutritionists and healthcare professionals is therefore important for the formulation of a tailor-made diet plan for an individual. Specific steps have thus been outlined for this purpose which may help both in meal planning and nutrition education.^29^

Efficacy of FEL also relies significantly on its cultural sensitivity and the availability of nutritional compositions; including total energy content especially the macro-nutrient content of traditional foods.^15^ Absence of such information places another challenge on the dietitians for managing the diet of their patients coming from different cultural backgrounds through the use of a “cultural sensitive diet plan”. Such plans may not be accurate or even possible in the absence of a FEL that confers with the specific culture.

Increased probability of adherence to specific diets regarding non communicable diseases have also been observed in the past using more culturally relevant FEL. Since it is observed that an individual chooses food for many reason and foods selected overtime can make a significant difference to the health of that individual.^30^

Diets planned from updated & locally relevant FELs have the benefit of greater acceptance with better chance of being implemented with success, thus aiding elimination of adherence related issues such as limited food items in the food exchange list, fewer options and unrealistic portion sizes.^24^

## CONCLUSION

The food exchange list is effective for managing chronic non-communicable diseases, for which dietary modification is a corner-stone of treatment. This tool helps to develop customized meals for people with little effort on their part as it provides extensive information regarding various food items in different groups i.e. serving sizes and nutrient values, thus facilitating the adherence to a diet prescribed as part of the medical nutrition therapy to effectively manage and prevent the reoccurrences of non communicable diseases; thus emphasizing the introduction of culturally relevant FELs to improve the nutritional status with increased probability of adherence for better management of NCDs.

### Authors’ Contribution

***MNK*** conceived, designed & manuscript writing.

***SK*** editing of manuscript.

***AAK*** did review and final approval of manuscript.

***MNK*** takes the responsibility and is accountable for all aspects of the work in ensuring that questions related to the accuracy or integrity of any part of the work are appropriately investigated and resolved.
